# Interstitial lung disease in a patient with anti-eIF2B antibodies-positive systemic sclerosis: A case report and literature review

**DOI:** 10.1016/j.rmcr.2024.102141

**Published:** 2024-11-22

**Authors:** Naoya Aoshiba, Kazutoshi Toriyama, Shohei Yamashita, Nao Shioiri, Yuko Iwata, Tomonori Uruma, Shinji Abe, Kenji Tsushima

**Affiliations:** a Department of Respiratory Medicine, Tokyo Medical University Hachioji Medical Center, 1163, Tatemachi, Hachioji-shi, Tokyo, Japan; b Department of Respiratory Medicine, Tokyo Medical University Hospital, 6-7-1 Nishishinjuku, Shinjuku-ku, Tokyo, Japan; c Department of Rheumatology, Tokyo Medical University Hachioji Medical Center, 1163, Tatemachi, Hachioji-shi, Tokyo, Japan

**Keywords:** anti-eIF2B antibodies, Interstitial lung disease, Systemic sclerosis

## Abstract

We report the case of a 76-year-old male patient with systemic sclerosis positive for anti-eukaryotic initiation factor 2B (eIF2B) antibodies. He presented to our hospital with dyspnea on exertion and, following a comprehensive physical examination, was diagnosed with interstitial lung disease associated with systemic sclerosis. Furthermore, systemic sclerosis was positive for the anti-eIF2B antibody. The presence of anti-eIF2B antibodies in systemic sclerosis is very rare, occurring in only 1–2.5 % of cases and seldom reported. Similar to our case, systemic sclerosis with positive anti-eIF2B antibodies has been reported to be more likely to be complicated by interstitial lung disease. Herein, we discuss our case in detail and summarize the previous findings.

## 1 Introduction

Systemic sclerosis (SSc) is an immunological disorder that causes fibrosis and microvascular damage to the skin and organs throughout the body [1]. Various autoantibodies are used to diagnose SSc, and identifying these autoantibodies is important because the complications and course of SSc can vary substantially depending on the type of autoantibody present. Although more than 90 % of autoantibodies in SSc are antinuclear antibodies, some cases test negative for these and instead present with anti-eukaryotic initiation factor 2 B (eIF2B) antibodies [2]. Anti-eIF2B antibodies were first identified as an autoantibody for SSc in 2016 [3], and there have been few reports of SSc associated with these autoantibodies [[3], [4], [5], [6], [7]]. Patients with anti-eIF2B antibodies-positive SSc are prone to developing interstitial lung disease (ILD) [5,8]; but the details are not well understood. We report a rare case of a patient diagnosed with anti-eIF2B antibody-positive SSc and determined to have ILD after a thorough examination.

## 2 Case report

A 76-year-old, male patient presented to our hospital with dyspnea on exertion. Six months ago, he consulted his family doctor about the dyspnea. He underwent a chest X-ray and was suspected of having interstitial lung disease (ILD). The ILD had not been closely investigated, and he was followed-up by his family doctor. He was referred to our hospital six months after his symptoms first appeared.

The dyspnea was classified as 2 by Hugh-Jones classification and was not accompanied by other symptoms, such as orthopnea, paroxysmal nocturnal dyspnea, chest pain or cough. He was aware of edema and Raynaud's phenomenon causing discoloration in his fingers from the same period. He had a history of gastroesophageal reflux disease, for which he was taking a proton pump inhibitor. He had no other medications, either currently or in the past. The patient was a current smoker and had a smoking history of 30 packs/year. His occupation was a school teacher. He had no history of exposure to fumes or dust, and he had never had pets. He had no family history of ILD.

**Fig. 1:**
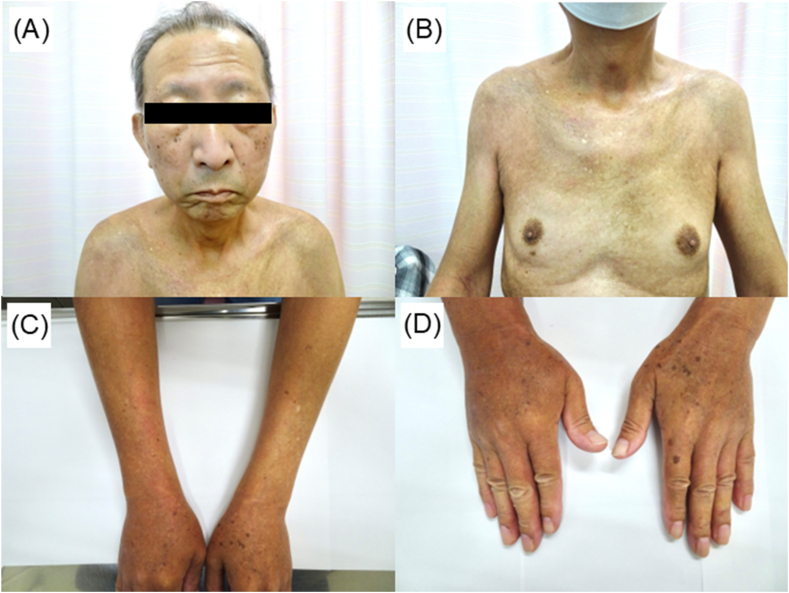
Skin thickening was observed on the face, chest, upper arms and fingers (A–D).

**Fig. 2:**
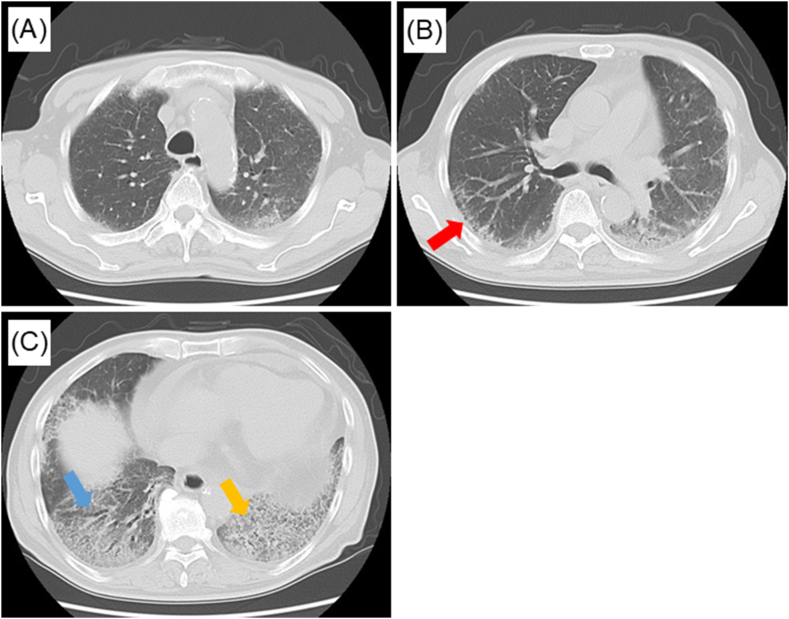
Chest computed tomography demonstrated subpleural reticular pattern (red arrow), traction bronchiectasis (blue arrow), and consolidation (yellow arrow) (A–C).

The patient's temperature was 36.3 °C, blood pressure was 111/70 mmHg, heart rate was 75/min, respiratory rate was 18/min, and SpO_2_ was 96 % on room air. Physical examination revealed skin thickening on the face, chest, upper arms, and fingers (Fig. 1A–D). The Rodnan score was 27: 2 for face, 2 for anterior chest, 1 for abdomen, 1 for both upper arms, 2 for both forearms, 2 for both hands, 3 for both fingers, 0 for both thighs, 1 for both legs, and 2 for both foots. There was no arthralgia or myalgia, and no neurological findings. Fine crackles ware predominantly heard in both lower back regions. Mild pitting edema of both lower legs was noted. Chest computed tomography during the inspiration phase demonstrated subpleural reticular pattern (red arrow), traction bronchiectasis (blue arrow), and consolidation (yellow arrow) (Fig. 2A–C). Pulmonary function tests indicated a reduced forced vital capacity of 2.13 L (63.7 % predicted) and diffusing capacity of lung for carbon monoxide (48.7 % predicted) (Table 1). Electrocardiography and echocardiography showed no evidence of pulmonary hypertension or heart dysfunction (Table 2). Systemic sclerosis (SSc) was strongly suspected; however, tests for anti-Scl-70 and anti-centromere antibodies were negative. Minor antibodies were also tested. Blood tests results were positive for anti-eIF2B and anti-SS-A/Ro52 antibodies, and the bronchoalveolar lavage fluid (BALF) showed an increased percentage of eosinophils (Table 3). α-streptococcus was cultured in small quantities, but were determined to be commensal bacteria. Based on these findings, the patient was diagnosed with ILD and diffuse cutaneous SSc, positive for anti-eIF2B antibodies. He was administered nintedanib and followed up at our hospital.

**Table 1:** Pulmonary function data by spirometry.

Parameter	Actual data	%predicted
VC (L)	2.11	61.3 %
ERV (L)	1.07	87.7 %
TV (L)	0.74	
IRV (L)	0.30	
IC (L)	1.04	
FVC (L)	2.13	63.7 %
FEV_1.0_ (L)	1.79	67.5 %
FEV_1.0_ % (%)	84.03	105.6 %
V_50_ (L/sec)	2.69	83.2 %
V_25_ (L/sec)	0.87	83.6 %
V_50_/V_25_	3.09	
RV (L)	1.00	61.0 %
TLC (L)	3.11	55.3 %
DL_CO_ (mL/min/mmHg)	6.81	48.7 %

VC: vital capacity, ERV: expiratory reserve volume, TV: total volume, IRV: inspiratory reserve volume, IC: inspiratory capacity, FVC: forced vital capacity, FEV1.0: forced expiratory volume 1.0 second, RV: residual volume, TLC: total lung capacity, DL_CO_: diffusing capacity of lung for carbon monoxide.

**Table 2:** Echocardiography.

Parameter	
LVEF	67 %
LA dilation	40 mm
TRV	2.57 m/sec
Estimated RVSP	31 mmHg
Estimated PADP	13 mmHg

LVEF: left ventricle ejection fraction, LA: left atrium, TRV: tricuspid regurgitation velocity, RVSP: right ventricle systolic pressure, PADP: pulmonary arterial diastolic pressure.

**Table 3:** Laboratory data.

Hematology				Immunology			
WBC	7990	4000–8000	/μL	IgG	1814	870–1700	mg/dL
Neut	66	40–60	%	IgA	284	110–410	mg/dL
Ly	18.5	25–45	%	IgM	153	33–190	mg/dL
Mo	12	3–7	%	ANA	40 >	40 >	times
Eo	2.6	1–6	%	Anti-Scl-70 Ab	(−)		
Ba	0.8	0–1	%	Anti-Cent Ab	(−)		
RBC	431	430–550	10^4^/μL	Anti-ARS Ab	(−)		
Hb	13.5	13.5–17.5	g/dL	Anti-MDA-5 Ab	(−)		
PLT	30.2	15–35	10^4^/μL	Anti-Mi-2 Ab	(−)		
				Anti-TIFI1-γ Ab	(−)		
**Biochemistry**				Anti-RNAPIII Ab	(−)		
TP	6.3	6.6–8.1	g/dL	Anti-RNAPI Ab	(−)		
Alb	3.2	4.1–5.1	g/dL	Anti-eIF2B Ab	144.9	0–10	
AST	28	13–30	U/L	Anti-SS-A/Ro Ab	377.2	0–13	
ALT	14	10–42	U/L	Anti-SS-B Ab/La	(−)		
LDH	283	124–222	U/L				
Cre	0.64	0.65–1.07	mg/dL	**Bronchoalveolar lavage fluid**			
BUN	18.2	8–20	mg/dL	Total cell count	149		10^3^/mL
Na	138	138–145	mEq/L	Neut	4.1		%
K	4.1	3.6–4.8	mEq/L	Ly	9.7		%
Cl	105	101–108	mEq/L	Mo	71.0		%
CRP	0.72	0–0.14	mg/dL	Eo	15.2		%
KL-6	1416	0–499	U/mL	Ba	0		%
BNP	45.0	0–18.4	pg/mL				

Test laboratory data and reference values are shown. WBC: white blood cell count, Neut: neutrophil, Ly: lymphocytes, Mo: monocytes, Eo: eosinophils, Ba: basophils, RBC: red blood cell count, Hb: hemoglobin, PLT: platelet, TP: total protein, Alb: albumin, AST: aspartate aminotransferase, ALT: alanine aminotransferase, LDH: lactate dehydrogenase, Cre: creatinine, BUN: blood urea nitrogen, Na: natrium, K: kalium, Cl: chlorine, CRP: c-reactive protein, KL-6: Krebs von den Lungen 6, ANA: anti-nuclear antibody, Anti-Scl-70 Ab: anti-scleroderma 70 antibody, Anti-Cent Ab: anti centromere antibody, Anti-ARS Ab: anti-aminoacyl-tRNA synthetase antibody, Anti-MDA5 Ab: anti-melanoma differentiation-associated gene 5 antibody, Anti-TIFI1-γ Ab: anti-transcriptional intermediary factor 1-γantibody, Anti-RNAPIII Ab: anti-ribonucleic acid polymerase III antibody, Anti-RNAPI Ab: anti-ribonucleic acid polymerase I antibody, Anti-eIF2B Ab: anti-eukaryotic translation initiation factor 2B antibody.

## 3 Discussion

We encountered a patient with systemic sclerosis positive for anti-eIF2B antibodies. The presence of anti-eIF2B antibodies in SSc is very rare. If SSc is strongly suspected but major antibodies are negative, minor antibodies should be tested.

Since the first report of SSc with anti-eIF2B antibody in 2016, 28 cases have been reported to date in our search [[3], [4], [5], [6], [7]]. The median age was 48 years, and 67.9 % of patients were female. Ninety-two point nine percent patients had ILD, and 71.4 % of them exhibited diffuse SSc (Table 4). However, as none of these were case reports, we did not have detailed information regarding these cases.

**Table 4:** Reports of systemic sclerosis with anti-eIF2B antibodies.

Patient	Median Age (years)	Female	ILD	Purmonary hypertation	Gastlic involvement	Renal disease	Diffuse SSc	Ref
7 cases	41 (38–52)	5	7	1	3	Unknown	6	3
3 cases	Unknown	2	3	1	Unknown	Unknown	2	4
3 cases	75 (65–88)	2	3	2	1	Unknown	2	5
9 cases	Unknown	6	8	1	8	1	8	6
6 cases	48 (36–62)	4	5	Unknown	Unknown	Unknown	3	7
Present case	76	0	1	0	1	0	1	

ILD: interstitial lung disease, SSc: systemic sclerosis, Ref: reference.

The eIF2B is a translation initiation factor consisting of five subunits (α to ε). It is present in the cytoplasm and regulates the initiation of protein synthesis [9]. Decreased eIF2B activity has been implicated in neurodegenerative diseases, metabolic syndromes, and cancer [10]. The frequency of anti-eIF2B antibodies in SSc is reported to be 1–2.5 % [[3], [4], [5], [6], [7]]. Molecular mimicry is one of the many hypothesized causes of SSc. Molecular mimicry refers to the structural similarity between a pathogen antigen and a host antigen that were originally unrelated, leading to an immune cross-reaction and causing autoimmune disease [10]. Additionally, SSc caused by anti-eIF2B antibodies can be explained by this molecular mimicry [11]. In 58 % of patients with SSc, IgG antibodies show abnormal reactivity to specific Epstein-Barr virus antigens, which may produce a protein similar to eIF2B, suggesting that Epstein-Barr virus molecular mimicry may target autoantigens and cause SSc [12].

We consulted a collagen specialist immediately after the patient's visit. In addition to SSc, mixed connective tissue disease and Sjögren's syndrome were initially considered as differential diagnoses in this case. After a thorough investigation of even minor antibodies, he was finally diagnosed with SSc. This case was characterized by a clear increase in eosinophils to 15 % in the BALF. No reports were found of evaluated eosinophil levels in the BALF of patients with SSc. The patient had no history of allergic disease, including asthma. It was more reasonable to consider ILD associated with SSc than eosinophilic pneumonia. However, the possibility that elevated eosinophil levels in BALF were due to the characteristic of SSc with a positive anti-eIF2b antibodies or drug-induced pneumonia caused by proton pomp inhibitor cannot be ruled out. Therefore, it is necessary to collect additional cases for further.

ILD was diagnosed with probable UIP pattern in the chest computed tomography. Pulmonary function tests indicated a reduced forced vital capacity. There is no absolute established treatment for ILD associated with SSc, and each patient's treatment should be individualized [13]. The collagen specialist determined that immunosuppressive drugs, such as mycophenolate and steroids, were not indicated in this case at this stage. Therefore, we administered nintedanib to the patient with ILD associated with SSc. A whole-body CT scan was performed as a screening, but no findings suspicious for cancer were observed. This is the first detailed case report of ILD caused by anti-eIF2B positive SSc, including imaging data, pulmonary function tests and BALF. The clinical presentation and course of this case did not differ significantly from those of common SSc. Reports of SSc positivity for anti-eIF2B antibodies should be collected.

## 4 Conclusion

If SSc is strongly suspected, even when anti-Scl-70 antibodies or anti-centromere antibodies are negative, minor antibodies should be investigated.

## CRediT authorship contribution statement

**Naoya Aoshiba:** Writing – original draft. **Kazutoshi Toriyama:** Writing – original draft. **Shohei Yamashita:** Writing – review & editing. **Nao Shioiri:** Writing – review & editing. **Yuko Iwata:** Writing – review & editing. **Tomonori Uruma:** Writing – review & editing. **Shinji Abe:** Writing – review & editing. **Kenji Tsushima:** Writing – review & editing.

## Declaration of competing interest

The authors declare that have no known competing financial interests or personal relationship that could have appeared to influence the work reported in this paper.
